# The Resting State of Taiwan EEG Normative Database: Z-Scores of Patients with Major Depressive Disorder as the Cross-Validation

**DOI:** 10.3390/brainsci13020351

**Published:** 2023-02-18

**Authors:** Yin-Chen Wu, I-Mei Lin

**Affiliations:** 1 Department of Psychology, College of Humanities and Social Sciences, Kaohsiung Medical University, Kaohsiung 807378, Taiwan; 2 Department of Medical Research, Kaohsiung Medical University Hospital, Kaohsiung 807378, Taiwan

**Keywords:** depression, electroencephalography (EEG), Taiwan EEG normative database, z-scores, quantitative electroencephalogram (qEEG)

## Abstract

This study referred to the standard of electroencephalography (EEG) collection of normative databases and collected the Taiwan normative database to examine the reliability and validation of the Taiwan EEG normative database. We included 260 healthy participants and divided them into five groups in 10-year age-group segments and calculated the EEG means, standard deviation, and z-scores. Internal consistency reliability was verified at different frequencies between the three electrode locations in the Taiwan normative database. We recruited 221 major depressive disorder (MDD) patients for cross-validation between the Taiwan and NeuroGuide normative databases. There were high internal consistency reliabilities for delta, theta, alpha, beta, and high-beta at C3, Cz, and C4 in the HC group. There were high correlations between the two z-scores of the Taiwan and NeuroGuide normative databases in the frontal, central, parietal, temporal, and occipital lobes from MDD patients. The beta z-scores in the frontal lobe and central area, and the high-beta z-scores in the frontal, central, parietal, temporal, and occipital lobes were greater than one for MDD patients; in addition, the beta and high-beta absolute value z-scores in the whole brain were greater than the ones of MDD patients. The Taiwan EEG normative database has good psychometric characteristics of internal consistency reliability and cross-validation.

## 1. Introduction

Several electroencephalography (EEG) normative databases are commercially available for clinical use, such as the Neurometrics database [1,2], BrainDx database [1], SKIL/SKIL3 database [3], and Neuroguide database (also called the Lifespan Normative EEG database; Applied Neuroscience, Inc.). Cuba, the Netherlands, and Karan also developed their EEG normative databases [4,5,6] (Table 1). The standard for the EEG normative database requires the following criteria: peer-reviewed publications, amplifier matching, artifact rejection, test re-test reliability, inclusion/exclusion criteria, adequate sample size per age group, appreciation for Gaussian, cross-validation, clinical correlation, and registration by the Food and Drug Administration (FDA) registered [7]. The criteria for establishing EEG normative databases usually include a large sample size, excluding clinically relevant diseases (physical or neurological illnesses or psychiatric disorders), covering a wide range of age groups (especially large sample sizes from children), and examining the effect of clinical application. This study reviewed the EEG normative database as follows.

The Neurometrics normative database was the first EEG normative database, and their EEG collection procedure was the same as the intelligence quotient (IQ) testing with the standardized test by using a large sample size [1]. The Neurometrics database excluded participants with head injuries, neurological or psychiatric disorders, psychological problems, alcohol or drug abuse, use of psychotropic medication, and academic or social problems. Finally, the Neurometrics database enrolled 782 healthy participants aged 6–90 years [1]. This normative database uses manual deartifacting and calculates artifact-free EEG from 0.5–25 Hz, including delta (1.5–3.5 Hz), theta (3.5–7.5 Hz), alpha (7.5–12.5 Hz), and beta (12.5–25 Hz), and then uses the natural logarithm to convert the EEG data to the Neuromertics normative database. EEG features included absolute power, relative power, coherence, mean frequency within the band, and symmetry for two minutes artifact-free. Different participants can compare with their age group normative database and then convert to a z-score to understand their relative position in the normative group. The BrainDX was based on the Neuromertics normative database and only included 16–80-year-old participants. BrainDX provides a normative database to discriminate clinical cases, such as Alzheimer’s-type dementia, depression, schizophrenia, learning disabilities, autism spectrum disorder, attention deficit hyperactivity disorder (ADHD), alcohol abuse, post-traumatic stress disorder, and post-concussive syndrome (https://braindx.net/research.php#application, accessed on 10 February 2023). The BrainDx has provided EEG parameters, including absolute power, relative power, mean frequency, intra- and inter-hemispheric symmetry, and intra- and inter-hemispheric coherence. 

Using the Neuromertics database, Deslandes et al. [8] enrolled 74 patients with comorbid dementia and depressive symptoms and 51 patients with comorbid major depressive disorder (MDD) and mild cognitive impairment, and analyzed two-minute artifact-free EEG. The results showed higher normed monopolar relative power theta at Cz, higher normed bipolar relative power theta for the head compared to the database, and lower normed monopolar relative power alpha for P3 compared to the database. The Neuromertics database can discriminate between the patient group and the healthy group and help understand the Neuromertics features of psychiatric disorders.

The Sterman–Kaiser imaging laboratory (SKIL) normative database was developed by Sterman and Kaiser [3] and excluded physical and psychiatric disorders and medication used. Finally, the SKIL database included 135 18–55-year-old healthy participants, 80% males (*n* = 108) and 20% females (*n* = 27). The sample comprised students and laboratory personnel (50%), community volunteers (25%), and United States Air Force personnel (25%). The new version of SKIL3 increased the sample size to 175 healthy individuals, including 115 adults, 30 teenagers (12–19 years), and 30 children (6–11 years) (adapted from https://bio-medical.com/media/support/skil3.pdf (accessed on 1 January 2023)). The application of SKIL, Siever and Collura [9] used SKIL for quantitative electroencephalogram (qEEG) evaluation for female college students with attention deficit disorder, and the results found a higher 7–8 Hz theta under audio-visual entrainment compared with the SKIL database.

The NeuroGuide normative database was developed by Thatcher et al. [7], and is registered with the United States FDA. This database included 625 healthy individuals aged two months to 82 years, 58.9% males and 41.1% females. The NeuroGuide normative database contains a multi-ethnic healthy population that includes 71.4% white, 24.2% black, and 3.2% oriental. Later, there was an increase of 155 healthy adults aged 14–82 years, and the total sample size increased to 727 [10]. The NeuroGuide database excludes disorders of consciousness, history of central nervous diseases, febrile or psychogenic convulsions, and abnormal deviation in mental and physical development. They also used the Wechsler Adult Intelligence Scale or the Wechsler Intelligence Scale for Children to assess participants’ IQ and exclude participants with abnormal IQ. In total, 943 EEG parameters were computed, including absolute power, relative power, coherence, phase, asymmetry, and power ratios. Regarding the application of the NeuroGuide database, Hammer et al. [11] compared the EEG data of insomnia to the NeuroGuide database, and found the EEG z-scores of absolute power, asymmetry, coherence, and phase were greater than the 1.96 standard deviation (SD) in insomnia patients, and then trained patients who had a z-score below 1.5 SD by using neurofeedback training.

The Cuba normative database was developed by Bosch-Bayard et al. [4], who collaborated with the Cuban government and the Ministry of Public Health of the Republic of Cuba. Healthy individuals with medical conditions, neurological diseases, psychiatric disorders, prenatal and perinatal antecedents, sleep disorders, familial pathological backgrounds, drug addiction, and abnormalities in the neurological physical examination were excluded. Finally, they collected 211 healthy participants (105 males and 106 females) from the Neurosciences Center of Cuba between 1988 and 1990, aged 5–80 years. Martínez-Briones et al. [12] compared children with learning disabilities with IQs higher than 75 with the Cuban normative database and found that children with learning disabilities had a z-score of theta/alpha ratio greater than 1.645.

The qEEG-Pro normative database (the qEEG-pro) was developed by Keizer et al. [5] in the Netherlands, the EEG was recorded by Deymed TruScanEEG (Deymed Diagnostic s.r.o., Czech Republic; http://www.deymed.com (accessed on 1 January 2023)) for 10 min of eyes-closed and eyes-open, excluded epileptiform activity and deleted EEG artifacts by using the Standardized Artifact Rejection Algorithm (S.A.R.A.; https:// www.qeeg.pro (accessed on 1 January 2023)). Finally, the qEEG-Pro database included 6–83-year-olds with 1482 eyes-closed EEG (955 males and 527 females) and 1232 eyes-open EEG (799 males and 432 females). The application of qEEG-Pro database, Bekker et al. [13] used BrainMaster with qEEG-Pro database to conduct infra-slow fluctuation neurofeedback training, revealing that qEEG was significantly improved within the standard range, and also improved the neurocognitive state and decreased depression, anxiety, and stress. 

The Korean normative database was developed by Ko et al. [6], who collected EEG data from the Korean EEG Center of Seoul National University. The participants’ cognitive function, emotional status, and behavioral problems were assessed to rule out psychiatric disorders (including behavioral or conduct disorders) or neurological diseases (including head trauma or epilepsy), and those who had histories of problematic academic or social activities were also excluded from the healthy normative database. In the end, 1289 healthy individuals (533 males and 736 females) were selected from the Imedisync Brain Normative Database (ISB-NormDB). After deleting movement and blink artifacts, the EEG data were converted to delta (1–4 Hz), theta (4–8 Hz), alpha (8–12 Hz), and beta (12–30 Hz), and log transformation was performed to avoid skewness problems. Using the Korean normative database, Ko et al. [6] used a male with attention deficit hyperactivity disorder (ADHD), a 78-year-old male with amnestic mild cognitive impairment (aMCI), and a female with anxiety to compare with the ISB-NormDB database. The results found greater theta power in ADHD patients with aMCI and greater beta3 power in anxiety compared to patients with the Korean normative database.

Most databases have not reported the mean and standard deviation (SD), the details of included and excluded criteria for collecting healthy participants, the standardized EEG collection procedure, or detailed background information (such as sample size for each age group, years of education, sex, and race). Even though the NeuroGuide normative databases contain a large sample size, the adult sample is only 167 and includes a small number of Chinese Americans (3.2%). Whether the NeuroGuide normative databases are suitable for Taiwanese participants has not yet been examined; whether Taiwan can develop its own normative database, and how consistent it is with the United States FDA-approved database needs to be examined.

The reasons for choosing the NeuroGuide database as the reference standard were: (1) A large sample size: 727 healthy individuals aged two months to 82.6 years [10,14]; (2) Participants with disorders of consciousness, central nervous system diseases, convulsions, and mental and physical disorders were excluded from the healthy population for EEG collection, and participants with abnormal IQs were also excluded; (3) Validity test: High correlations in z-scores of the delta, theta, alpha, and beta (*r* = 0.757–0.979) were confirmed between the NeuroGuide database (Applied Neuroscience Inc., Seminole, Florida) and BrainDx database (BrainMaster Technologies Inc., Bedford, Ohio) [15], and the high correlations in the z-scores of 1–30 Hz at eyes-open and eyes-closed (*r* = 0.94) were confirmed between the NeuroGuide database and qEEG-Pro database [5]; (4) EEG recording and signal processing: most of the normative databases measured EEG in the resting state, and manually deartifacting, such as the NeuroGuide database [2,4,5,6,7], few normative databases analyzed by automatic, independent component analysis, or principal component analysis to avoid data distortion [5,16]; (5) Reliability and validity: this research used the same group of participants to measure EEG at different time points to verify the consistency between the two EEG measurements [7]. Two validations were performed in the normative database. First, the completely independent cross-validation used the third group of samples as the benchmark, calculating the z-scores from the two normative databases and then calculating the correlation coefficients of the two z-scores to obtain an index of consistency. Thatcher and Lubar [15] collected a third cohort sample with 332 mental disorders, with participants aged 6.2–84.9 years to verify the correlations between the NeuroGuide normative database and the Neurometric normative database, revealing high correlations of delta, theta, alpha, and beta between the two z-scores (*r* = 0.757 to *r* = 0.979), with good cross-validation. Second, leave-one-out cross-validation removed one participant from the distribution and calculated the z-scores of all EEG parameters with the mean and SD of the age group. This process was repeated for each participant, and the z-score distribution for each EEG frequency band was tabulated for each participant. Completely independent cross-validation is a more rigorous method, but it is more expensive and difficult; therefore, most databases choose to use leave-one-out cross-validation [16]. Therefore, the above information shows that the NeuroGuide database has strict inclusion and exclusion criteria for EEG collection, including sufficient samples across different ages, sexes, and races, and also has good psychometric characteristics of internal consistency and cross-validation, which can be used as the reference standard of the Taiwan normative database (Table 1).

The main purpose of the normative database is to quantify an individual’s EEG differences and define their EEG relative position in the normative group. The most common method is to compare a group or specific individual with a healthy normative database and convert it to a z-score to obtain the relative position in the group. A Gaussian distribution of EEG parametric statistics assumed a normal distribution; 68.26% of the individuals fell within the range of the mean ± 1 SD, 95.44% of the individuals fell within the range of the mean ± 2 SD, and 99.74% of the individuals fell within the range of the mean ± 3 SD. While a z-score of zero indicates that the EEG has more flexibility and adaptability to the external environment, z-scores higher than ± 1 indicate potential abnormalities [17]. Therefore, the normative database can be used to detect and distinguish abnormal EEG values of clinical patients and can also be used as a reference for planning neurofeedback training, as well as to compare the improvement of neurofeedback before and after training [14]. Therefore, the normative database can convert EEG data to different z-scores, including absolute power, relative power, power ratio, asymmetry, coherence, and phase [17]. 

Previous studies have found that brain hyperactivity in MDD patients with comorbid anxiety symptoms [18,19,20,21], can be a biomarker for MDD. Other studies also confirmed higher beta activity in the frontal, central, parietal, and occipital lobes in MDD patients than in healthy controls [22,23,24]. Moreover, beta activity has been correlated with depression and anxiety symptoms in MDD patients [25,26,27,28]. This study aimed: (1) To develop the EEG normative database of healthy populations aged 20–70 years in Taiwan. The mean and SD of each EEG frequency band were calculated in 10-year age-group segments; (2) To perform cross-validation between the Taiwan normative database and the NeuroGuide normative database; (3) To examine the correlations between symptoms of depression and anxiety and EEG z-scores in MDD patients.

## 2. Materials and Methods

### 2.1. Participants

Participants were recruited from 2013 to 2022 in Kaohsiung City, Taiwan, and included a healthy control group (HC group) and MDD patients comorbid with anxiety disorders or with anxiety symptoms (MDD group).

**Figure 1:**
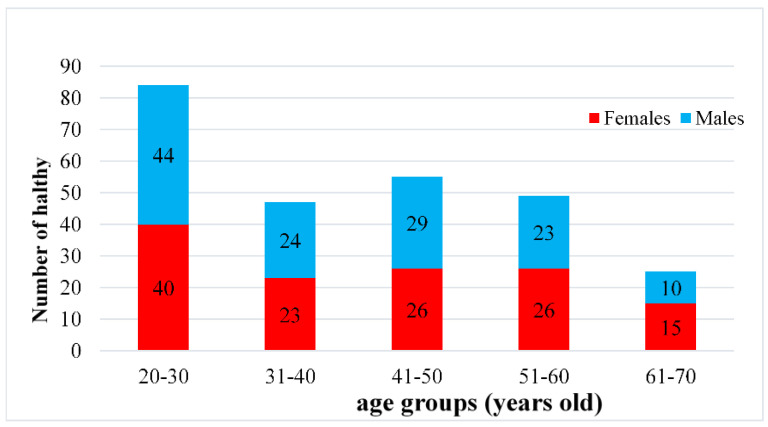
Number of healthy participants of the Taiwan normative database.

The HC group was recruited from the Health Management Center of Kaohsiung Medical University Hospital, Kaohsiung Medical University campus, and the Kaohsiung city community. The inclusion criteria were as follows: (1) Participants were referred by doctors or nurses in the health management center and were confirmed to be without physical illnesses (e.g., cancer, kidney disease, or stroke) or psychiatric disorders (e.g., depressive disorder, anxiety disorder, bipolar disorder, or schizophrenia), their health examination reports were normal, and they did not take any prescribed medication; (2) Participants were self-enrolled by study flyers, that instructed participants to provide their latest year’s health examination report and confirm the absence of physical illnesses or psychiatric disorders and without taking any prescribed medication; (3) The total scores of the Beck Depression Inventory-II (BDI–II) and Beck Anxiety Inventory (BAI) were lower than 14 and 8, respectively, which were measured by researchers; (4) Participants had to be aged 20–70 years. The age range was divided into 10-year age-group segments (20–30, 31–40, 41–50, 51–60, and 61–70 years) for five age groups; each group included at least 15 females and 15 males for a normative database (Figure 1). However, only 10 males in the 61–70 age group were in the healthy control group due to physical and mental conditions.

We recruited 296 healthy participants, and excluded 17 (age < 20 years [*n* = 1], BDI-II or BAI higher than 14 or 8 [*n* = 11], with physical illnesses [*n* = 5]). In total, 279 healthy participants completed the EEG measurement, and 19 healthy participants had their EEG data deleted from the statistical analysis (EEG artifacts [*n* = 10] and damaged EEG data [*n* = 9]. Finally, 260 healthy participants were included in the HC group (130 males and 130 females). 

The inclusion criteria for the MDD group were as follows: (1) MDD patients diagnosed by psychiatrists based on the Diagnostic and Statistical Manual of Mental Disorders 5th Edition (DSM−5; American Psychiatry Association, 2003). MDD patients who had comorbid anxiety disorders or anxiety symptoms were permitted according to the primary diagnosis of MDD; (2) The total scores of the Beck Depression Inventory-II (BDI–II) and Beck Anxiety Inventory (BAI) were higher than 14 and 8, respectively; (3) Participants’ age was between 20 and 70 years. Exclusion criteria were as follows: (1) MDD comorbid with other psychiatric disorders (e.g., bipolar disorder, substance use, and schizophrenia) or other severe physical illnesses (e.g., dementia, cancer, kidney disease, and stroke). 

In total, 333 participants with MDD were referred by psychiatrists from three medical centers in Kaohsiung city, after excluding 97 participants (age < 20 [*n* = 8], BDI-II and BAI not higher than 14 and 8 [*n*=78], the primary diagnosis was not MDD [*n* = 2], or comorbid other illnesses [*n* = 9]). There were 236 participants in the MDD group who completed the EEG measurement and 15 participants whose EEG data were deleted from the statistical analysis (EEG artifacts [*n* = 13] and EEG data damaged [n=2]). Finally, there were 221 participants (163 females and 58 males) in the MDD group. 

Institutional review board approval was obtained from the ethics committees of Kaohsiung Medical University Hospital, Taiwan (KMUH-IRB−2012−02−09-II, KMUH-IRB-F-I−20160027, and KMUHIRB-F(I)−20200117) and Kaohsiung Chang Gung Memorial Hospital, Taiwan (CGMH IRB:1604250002). Each participant provided informed consent and received NT 500 (approximately USD 16) for their participation. 

### 2.2. Material

(1) Psychological questionnaires: Demographic data (e.g., age and sex), BDI-II, and BAI were measured. The BDI-II includes 21 items scored on a four-point Likert scale to assess depressive symptoms. The total score of the BDI–II ranges from 0 to 63, and a higher BDI-II score indicates more depressive symptoms [29]. The Chinese version of the BDI–II was translated by Chen [30], where Cronbach’s α was 0.94, split-half reliability was 0.91, and the BDI-II correlated with the Chinese Health Questionnaire was 0.69 [31] (Lu et al., 2002). The BAI includes 21 items scored on a four-point Likert scale to assess anxiety symptoms. The total score ranges from 0 to 63, with higher BAI scores indicating more anxiety symptoms [32] (Beck et al., 1988). The Chinese version of the BDI–II was translated by Lin [33], where Cronbach’s α was 0.95, the split-half reliability was 0.91, and the BAI correlated with the Hamilton Anxiety Scale was 0.72 [34]. The BDI-II and BAI, which have good psychometric characteristics, were used to assess depression and anxiety in clinical practice;
(2) EEG recording: EEG raw signals were recorded using the BrainMaster Discovery 24 with impedance lid amplifier equipment and BrainAvatar 4.0 software (BrainMaster Technologies, Inc., Bedford, Ohio). A 19-channel EEG cap (Electro-Cap International, Inc., Eaton Ohio) based on the international 10−20 system, including Fp1, Fp2, Fz, F3, F4, F7, F8, Cz, C3, C4, T3, T4, T5, T6, Pz, P3, P4, O1, and O2, was connected to the BrainMaster equipment to measure the raw EEG signals. A linked-ear reference, impedance below 5 kΩ, 0–100 Hz of the bandpass filter of 60 Hz, notch filter, and sampling rate of 256 Hz were applied during the EEG recording.

The following instructions were given to participants the day before the EEG recording: (a) Keep a normal routine and do not stay up the night before the EEG recording; (b) Wash your hair to clean the scalp the day before the EEG recording and do not use a hair conditioner to avoid high impendence. Do not use hair gel or other beauty products after shampooing; (c) Abstain from alcohol, coffee, and tea three hours before the EEG recording. The EEG was recorded between 9 am and 5 pm. All participants were instructed to sit comfortably and rest in a temperature-controlled room (between 24–28 °C), and the EEG signals were recorded for five minutes with eyes closed in the resting state.

### 2.3. Data Reduction, EEG Processing, and Statistical Analysis

The EEG data were analyzed using Neuroguide software version 3.2.6.0 (Applied Neuroscience, Inc., FL, USA). The researchers checked the EEG artifacts by setting up a 20-s window and removing movement and eye-blinking artifacts. At least 60 s of EEG data were saved and then transferred to absolute EEG power by the Fast Fourier Transform at the following frequency bands: delta (1–4 Hz), theta (4–8 Hz), alpha (8–12 Hz), beta (12–25 Hz), and high-beta (25–30 Hz) in the frontal (F3, F4, Fz), central (C3, C4, Cz), parietal (P3, P4, Pz), temporal (T3, T4, T5, T6), and occipital lobes (O1, O2). 

Since the mean and SD of the NeuroGuide database could not be obtained, we used the EEG absolute power of the MDD group compared to their age group with the Taiwan normative database and the NeuroGuide normative database, and then converted these EEG absolute powers into z-scores and absolute value z-scores (|z score|). The correlations between the two z-scores were calculated as a cross-validation of the Taiwan normative database.

Data reduction included the following steps: (1) This study calculated the mean and SD of the delta, theta, alpha, beta, and high-beta in five age groups for 260 participants in the HC group, including 20–30, 31–40, 41–50, 51–60, and 61–70 age groups; (2) The z-scores and absolute value z-scores were calculated using the equation (*z* = (x–μ) /σ) for the MDD group for each discrete EEG frequency band: delta, theta, alpha, beta, and high-beta. The total number of z-scores for healthy participants was 260 × 19 electrodes × 5 frequency bands × 5 discrete aged group = 123,500 z-scores, and the total number of z-scores for the MDD group was 221 × 19 electrodes × 5 frequency bands × 5 discrete aged group = 104,975 z-scores; (3) The z-scores and absolute z-scores in the MDD group were calculated for each EEG frequency band at different locations. 

All statistical analyses were performed using SPSS Statistics 21.0 (IBM Corporation, Armonk, NY, USA), and Student’s *t*-test and chi-square (*χ*^2^) were used to examine group differences in demographic data and psychological questionnaires. The R software version 4.2.0 (Free Software Foundation, Boston, MA) with the ggplot2 package was used to redraw the EEG absolute power with locally weighted scatterplot smoothing method. One-way analysis of variance (ANOVA) was used to compare the EEG parameters between five age groups. The Mauchly sphericity test with Greenhouse–Geisser adjustment was applied in one-way ANOVA, and the Bonferroni method was used for post hoc comparisons. This study used internal consistency and independent cross-validation to examine the reliability and validity of the Taiwanese normative database. The Cronbach’s alpha of delta, theta, alpha, beta, and high-beta at C3, C4, and Cz for the Taiwan normative database were used to present internal consistency. The MDD group was the third group for the benchmark, and their EEG data were compared to the Taiwan and NeuroGuide normative databases. Pearson correlation coefficients were analyzed for z-scores between the Taiwan normative database and the NeuroGuide normative database for the MDD group to confirm completely independent cross-validation. Finally, the Pearson correlation coefficients were analyzed for depression, anxiety, z-scores, and absolute value z-scores in the MDD group.

## 3. Results

### 3.1. Participants’ Demographic Characteristics between the HC Group and the MDD Group

We included 260 participants in the HC group and 221 in the MDD group; no significant difference was found in age between the two groups (*t*_(479)_ = –0.35, *p* > 0.05). The two groups significantly differed in terms of sex (*χ*_(1)_^2^ = 28.32, *p* < 0.001). In the cell comparison, males and females did not significantly differ in the HC and MDD groups; however, there were more females in the MDD group than in the HC group, as well as fewer males in the MDD group than in the HC group. Moreover, higher total scores of depression and anxiety were observed in the MDD group than in the HC group (*t*_(261.92)_ = –37.47, *p* < 0.001; *t*_(234.21)_ = –30.00, *p* < 0.001, respectively; Table 2). 

**Figure 2:**
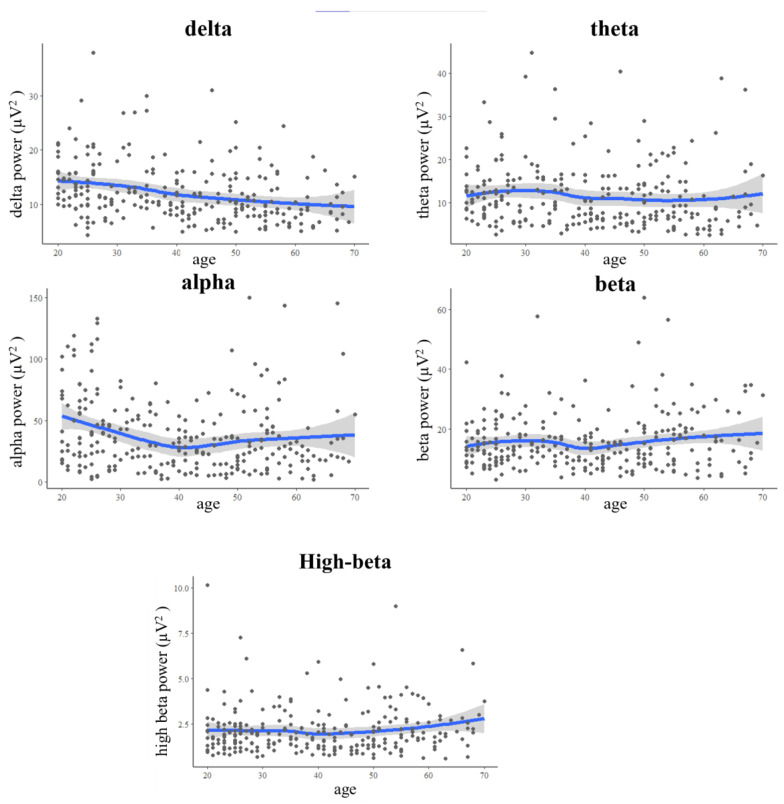
The EEG absolute power across different age groups. Note: The blue lines are locally weighted scatterplot smoothing of EEG absolute power. The gray areas are 95% of the confidence interval for EEG absolute power. The black dots are scatterplots of EEG absolute power for each participant.

Figure 2 shows the EEG frequency bands (delta, theta, alpha, beta, and high-beta) across the 20–70 age groups. Slow waves (delta, theta, and alpha) showed a decreasing trend with age, but fast waves (beta and high-beta) showed a slight increasing trend after 50 years of age. One-way ANOVA showed a significant difference in the delta between the five age groups (*F* = 5.929, *p* < 0.001), and the post hoc comparison with Bonferroni found that healthy participants in the 20–30 age group had higher delta compared with the 51–60 and 61–70 age groups (*p* < 0.002 and *p* < 0.015, respectively), as well as that the 31–40 age group had a higher delta than that in the 51–60 age group (*p* < 0.020). Therefore, young healthy participants have higher delta than older healthy participants. Moreover, females had higher EEG parameters than males in the 20–30, 51–60, and 61–70 age groups.

### 3.2. Reliability and Validity in the Taiwan Normative Database

The internal consistencies of Cronbach’s alpha for delta, theta, alpha, beta, and high-beta at Cz, C3, and C4 were 0.936, 0.981, 0.985, 0.986, and 0.917 for the HC group, respectively. The results demonstrate the high internal reliability of the Taiwan normative database.

For the cross-validation, the z-scores for delta, theta, alpha, beta, and high-beta significantly correlated between the Taiwan normative database and the NeuroGuide normative database in the frontal, central, parietal, temporal, and occipital lobes in the MDD group. The correlation coefficients were *r* = 0.707 (*p* < 0.001) and *r* = 0.915 (*p* < 0.001; Table 3).

### 3.3. The Z-Scores and Absolute Value Z-Scores in the MDD Group

**Figure 3:**
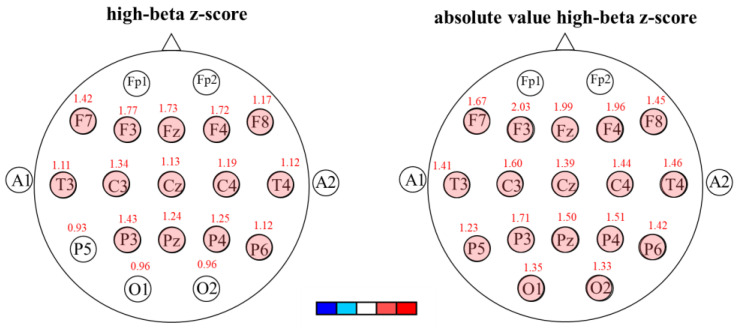
The high-beta z-scores (**left**) and high-beta absolute value z-scores (**right**) in the MDD group.

This study converted the raw EEG data of the MDD group and compared them to the Taiwan database for each age group; the z-scores and absolute value z-scores are presented in Table 4. Participants in the MDD group had beta z-scores greater than 1SD in the frontal (Fz/F3/F4/F7) and central (Cz), as well as high-beta z-scores greater than 1SD in the frontal (Fz/F3/F4/F7/F8), central (Cz/C3/C4), parietal (Pz/P3/P4), and temporal lobes (T3/T4/T6) (Figure 3). Moreover, the beta and high-beta absolute value z-scores were greater than 1SD in the frontal (Fz/F3/F4/F7/F8), central (Cz/C3/C4), parietal (Pz/P3/P4), temporal (T3/T4/T5/T6), and occipital lobes (O1/O2) (Table 4).

### 3.4. Correlations between Depression, Anxiety, and Z-Scores in the MDD Group

Somatic anxiety, total score of anxiety, and beta at the parietal lobe positively correlated with the temporal lobe, as did cognitive depression, somatic depression, total score of depression, somatic anxiety, and high-beta, especially in the frontal, parietal, and temporal lobes. Somatic depression negatively correlated with delta at F4 and T4 (*r* = –0.15, *p* < 0.05; and *r* = –0.14, *p* < 0.05, respectively), as did cognitive anxiety, total anxiety score, and delta at T3 (*r* = –0.16, *p* < 0.05; and *r* = –0.16, *p* < 0.05, respectively) (Appendix A, Table A1).

## 4. Discussion

This study collected EEG data from healthy participants with a large sample size and established the mean and SD of ten age groups from 20 to 70 years of age as the Taiwan normative database. In total, 260 healthy participants were recruited for this study, and each age group included more than 30 participants, half males and half females. The number of healthy participants aged higher than 20 years exceeded the SKIL3 databases [3] and NeuroGuide databases [7]. Figure 2 presents the scatterplots of the delta, theta, alpha, beta, and high-beta absolute power for each age group, and the trends are similar to those in the Korean and Cuban databases [4,6].

In this study, the EEG raw scores of the MDD group were compared with the Taiwan database and the NeuroGuide database and then converted to the z-scores and absolute value z-scores for each EEG frequency band. The z-scores have high correlations between the Taiwan database and the NeuroGuide database (*r* = 0.71–0.92). This was consistent with Keizer [5], who compared the z-scores between the qEEG-Pro database and the NeuroGuide database, and found that the z-scores correlations in each frequency band were between *r* = 0.89–0.97. Although most correlations in this study were over *r* = 0.8, some z-scores were as high as r = 0.9 or more. However, two of the z-scores had slightly lower correlations in the alpha band at O1 and O2 (*r* = 0.71 and *r* = 0.76), possibly because the EEG measurement was conducted in a resting state with eyes closed. Although the EEG of MDD patients were compared with the eyes-closed state of the NeuroGuide database, possibly because some participants were too relaxed and sleepy, under antidepressant prescription, or different EEG measurement situations between the Taiwan and NeuroGuide databases, there was more alpha in the occipital lobe. We strictly controlled the measurement situation, such as the indoor temperature, laboratory setting, and EEG measurements during the daytime. However, few MDD patients were measured in the hospital between 1:00–3:00 pm, maybe in the resting state with eyes-closed being too relaxed and asleep.

Both beta z-score in the frontal and central areas and high-beta z-scores in the frontal, central, parietal, and temporal lobes were greater than 1SD; the means of beta and high-beta absolute value z-scores were both greater than 1SD in the frontal, parietal, temporal, and occipital lobes, and the central area in the MDD group. These results support brain hyperactivity in patients with MDD [35] and are consistent with those of previous studies [18,19,36,37,38]. Some studies have found a higher beta in the parietal and occipital lobes (P3, P4, O1, and O2) [24] and a higher beta in the frontal lobe [24,39,40]. A review of 18 studies also confirmed a higher beta band in the frontal lobe for patients with MDD [41]. In addition, our study found a lower delta in the MDD group, and Mumtaz et al. [42] also reported a lower delta in the frontal and occipital lobes in MDD patients compared to those in healthy controls.

Some studies reported a frontal alpha asymmetry in patients with MDD [43,44], and a study found higher activation in right anterior and posterior brain regions compared to the left sides in patients with anxious MDD [36]. However, our study did not find higher z-scores or absolute value z-scores in the frontal, temporal, parietal, central, and occipital regions in patients with MDD. Our study supported brain hyperactivity over the brain regions [18,19]. Brain hyperactivity may be a clinical biomarker for distinguishing patients with MDD from healthy participants in the future. Therefore, the Taiwan normative database for quantitative EEG (QEEG)-based assessment and neurofeedback intervention will be developed to examine the neurofeedback efficacy in decreasing clinical symptoms and improving EEG parameters in the future.

Higher depression and somatic anxiety in the frontal, parietal, and temporal lobes were related to higher beta and high-beta activity. This is consistent with the results of previous studies; Pizzagalli et al. [26] found that depressive symptoms (Beck Depression Inventory) and anxiety (State-Trait Anxiety Inventory) were positively related to right frontal beta3 (21.5–30.0 Hz) in MDD patients; Saletu et al. [27] found that depressive symptoms, which were measured by the Hamilton Depression Rating Scale, were related to beta4 in depressed menopausal syndrome patients. Sachs et al. [28] found that higher beta was positively related to the State-Trait Anxiety Inventory score in patients with social anxiety disorder. Lee et al. [25] found that higher anxiety scores on the Hamilton Anxiety Rating Scale were related to beta. These studies have supported the theories of brain hyperactivity in patients with anxious MDD [18,19,36,37,38]. Moreover, higher somatic depression and cognitive anxiety were related to a lower delta in the frontal and temporal lobes in the MDD group, indicating that higher depressive and anxiety symptoms were related to brain hyperactivity and caused higher beta and high-beta and lower delta in MDD patients.

This study had several limitations. First, the Taiwan normative database of healthy participants is half male and half female, and each age group included more than 30 healthy participants. However, more females than males were found in the MDD group than in the HC group. Although these situations were matched for the prevalence of MDD [45], a case–control study design and matched sex would be suitable for future studies. Second, higher educational levels were found in the HC group than in the MDD group. Although this situation was consistent with a large sample size study [46], a matched education level between the HC and MDD groups would be suitable for future study. Third, the prevalence of physical illness increases with age, it limited the healthy old participants who were recruited. The sample size can be accumulated in future studies to increase the representativeness of the Taiwan normative database.

## 5. Conclusions

The Taiwan EEG normative database has good psychometric characteristics with an internal consistency reliability and cross-validation between the Taiwan normative database and the US normative database. The Taiwan EEG normative database will be applied to clinical practice in future studies.

## Figures and Tables

**Table 1:** The EEG normative database.

		John [1]	John [2]	Sterman & Kaiser [3]	Thatcher [7]	Bosch-Bayard [4]	Keizer [5]	Ko [6]	This Study
Country		USA	USA	USA	USA	Cuban	Netherlands	Koran	Taiwan
Name of database		Neurometrics	BrainDx	SKIL/SKIL3	NeuroGuide		qEEG pro	ISB-NormDB	
FDA-approved		Yes	Yes	No	Yes	No	Yes	Yes (KFDA)	No
Years of data collection					1979−1987, 2000	1988−1990	2004−2013	2014−2019	2012−2022
Sample size		782	464	135 SKIL/ 175 SKIL3	EC625/EO625 in 2003; EC727/EC727 in 2013	211	EC1482/ EO1232	1289	260
Age (y)		6−90	16−80	18−55	2 months−82.6	5−80	6−83	4.5−81	20−70
Sex (M/F)				108M/27F for SKIL	355M/270F	105M/106F	EC:955/527 EO:799/432	553M/ 736F	130M/ 130F
Children, *n* (years old)		356 (6−16)	310	30 (6−11) SKIL3	458 (6−16)				0
Adults, *n* (years old)		426 (16−90)	154 (16−80)	135(18−55) SKIL/115 adults (20−75), 30 teenagers SKIL3	167(16−82)/ 155(14−82)				260 (20−70)
EEG placement		10−20	10−20	10−20	10−20	10−20	10−20	10−20	10−20
Channels		19	19	19	19	19	19	19	19
Reference		Linked-ear	Linked-ear	Linked-ear	Linked-ear	Monopolar linked ear		Linked-ear	Linked-ear
Sampling rate (Hz/s)		256	≥ 100			256	128(8%) & 256(92%)	250	256
Impedance (KΩ)		5	5	5	5,10	5		5	5
EC/EO		EC	EC	EC/EO/Task	EC/EO	5EC/3EO/ 3HV	EC/EO	4EC/4EO	EC
Recording time		20−30 m resting state	20−30 m resting state	2−4 m resting state	58.6 s−40 m	10−12 m	10 m resting state	8 m resting state	5 m resting state
Frequency bands (Hz)		0.5−25	0.5−50	1−20	0.3−30	0.5−30	1−45	1−45.5	1−40
	delta	1.5−3.5	1.5−3.5	1−3	1−3.5	1−4	1−3	1−4	1−4
	theta	3.5−7.5	3.5−7.5	3−7	3.5−7.5	4−8	4−7	4−8	4−8
	alpha	7.5−12.5	7.5−12.5	7−12	7.5−12.5	8−12	8−12	8−12	8−12
	beta	13.5−25	13.5−25	12−15	12.5−19	12−25	13−20	12−30	12−25
	high-beta		Beat 2:25−35	15−20		25−30	21−30		25−30
Deartifacting		Manual	Manual	Manual/ Automatic	Manual	Manual	Automatic	Automatic	Manual

Note: EC, eyes-closed; EO, eyes-open; F, female; FDA, United State Food and Drug Administration; HV, hyperventilation; M, male.

**Table 2:** The demographic characteristic between the HC group and MDD group.

		HC Group (*n* = 260)	MDD Group (*n* = 221)		
		M (SD)	M (SD)	*t /χ^2^*	*p*
Age, years old		40.63 (14.11)	41.09 (14.02)	*t =* −0.35	0.726
20–30, *n* (F/M)		84 (40/44)	65 (41/24)		
31–40, *n* (F/M)		47 (23/24)	39 (33/6)		
41–50, *n* (F/M)		55 (26/29)	55 (43/12)		
51–60, *n* (F/M)		49 (26/23)	37 (28/9)		
61–70, *n* (F/M)		25 (15/10)	25 (18/7)		
Sex	Females (F)	130 ^a^	163 ^b^	*χ ^2^ =* 28.32 ***	<0.001
	Males (M)	130 ^a^	58 ^b^		
Education	Primary school	2 (0.76%) ^a^	6 (2.71%) ^a^	*χ ^2^ =* 61.21 ***	<0.001
	Junior high school	4 (1.54%) ^a^	20 (9.05%) ^b^		
	Senior/vocation high school	32 (12.31%) ^a^	66 (29.86%) ^b^		
	Junior college	33 (12.69%) ^a^	32 (14.48%) ^a^		
	University	113 (43.46%) ^a^	77 (34.84%) ^a^		
	Graduate school	76 (29.23%) ^a^	20 (9.05%) ^b^		
BDI-II		4.23 (3.52)	31.93 (10.50)	*t =* −37.47 ***	<0.001
BAI		1.95 (1.93)	22.28 (9.91)	*t =* −30.00 ***	<0.001

*** *p* < 0.001. Note: BDI-II, Beck Depression Inventory-II; BAI, Beck Anxiety Inventory; F, females; M, males. ^a^ no significant difference between the HC and the MDD groups; ^b^ significant differences between the HC and the MDD groups

**Table 3:** The Pearson’s correlations of z-scores between the Taiwan and NeuroGuide databases.

	Frontal Lobe					Central Area			
	Fz	F3	F4	F7	F8	Cz	C3	C4	
delta	0.83 ***	0.89 ***	0.88 ***	0.81 ***	0.88 ***	0.90 ***	0.90 ***	0.90 ***	
theta	0.84 ***	0.85 ***	0.84 ***	0.83 ***	0.85 ***	0.86 ***	0.85 ***	0.86 ***	
alpha	0.85 ***	0.85 ***	0.85 ***	0.87 ***	0.85 ***	0.84 ***	0.84 ***	0.83 ***	
beta	0.86 ***	0.88 ***	0.89 ***	0.89 ***	0.91 ***	0.85 ***	0.87 ***	0.88 ***	
high-beta	0.87 ***	0.89 ***	0.91 ***	0.84 ***	0.87 ***	0.90 ***	0.89 ***	0.83 ***	
	Parietal lobe			Temporal lobe			Occipital lobe		
	Pz	P3	P4	T3	T4	T5	T6	O1	O2
delta	0.90 ***	0.92 ***	0.88 ***	0.87 ***	0.86 ***	0.89 ***	0.87 ***	0.82 ***	0.88 ***
theta	0.85 ***	0.85 ***	0.87 ***	0.88 ***	0.89 ***	0.82 ***	0.89 ***	0.86 ***	0.88 ***
alpha	0.82 ***	0.79 ***	0.82 ***	0.85 ***	0.83 ***	0.72 ***	0.80 ***	0.71 ***	0.76 ***
beta	0.87 ***	0.86 ***	0.89 ***	0.90 ***	0.90 ***	0.86 ***	0.88 ***	0.83 ***	0.85 ***
high-beta	0.89 ***	0.88 ***	0.90 ***	0.84 ***	0.83 ***	0.92 ***	0.91 ***	0.86 ***	0.89 ***

*** *p* < 0.001.

**Table 4:** The z-scores and absolute value z-scores in the MDD group.

Z-Scores					
	Delta	Theta	Alpha	Beta	High-Beta
	M (SD)	M (SD)	M (SD)	M (SD)	M (SD)
Fz	−0.11(1.41)	−0.03(1.30)	0.07(1.22)	1.22(2.45)	1.73(3.05)
F3	−0.08(1.13)	−0.02(1.24)	0.09(1.23)	1.21(2.42)	1.77(2.97)
F4	−0.17(1.04)	−0.03(1.30)	0.06(1.24)	1.26(2.54)	1.72(2.89)
F7	−0.08(1.12)	−0.06(1.12)	0.06(1.14)	1.02(1.94)	1.42(2.43)
F8	−0.15(1.01)	−0.10(1.17)	0.03(1.21)	0.90(1.98)	1.17(2.22)
Cz	−0.18(0.99)	−0.06(1.20)	0.05(1.23)	1.12(2.48)	1.13(2.10)
C3	−0.15(1.00)	−0.01(1.23)	0.08(1.26)	0.86(1.93)	1.34(2.44)
C4	−0.16(1.02)	0.00(1.29)	0.11(1.25)	0.88(2.00)	1.19(2.46)
Pz	−0.09(1.08)	−0.05(1.00)	0.02(1.16)	0.72(1.99)	1.24(2.39)
P3	−0.11(1.08)	−0.04(1.03)	0.05(1.25)	0.67(1.84)	1.43(2.93)
P4	−0.09(1.10)	−0.02(1.03)	0.06(1.23)	0.70(1.87)	1.25(2.41)
T3	−0.20(0.89)	−0.04(1.20)	0.12(1.24)	0.95(1.91)	1.11(2.16)
T4	−0.19(1.20)	−0.11(1.17)	0.06(1.25)	0.93(2.08)	1.12(2.34)
T5	−0.16(0.97)	−0.03(1.28)	0.09(1.53)	0.67(1.78)	0.93(1.85)
T6	−0.10(1.04)	0.03(1.25)	0.14(1.52)	0.95(2.33)	1.12(2.01)
O1	−0.02(1.59)	0.00(1.04)	0.08(1.46)	0.67(1.91)	0.96(2.19)
O2	−0.08(0.94)	−0.03(0.89)	0.07(1.24)	0.57(1.61)	0.96(2.12)
Absolute value z-scores					
	delta	theta	alpha	beta	high-beta
	M (SD)	M (SD)	M (SD)	M (SD)	M (SD)
|Fz|	0.86(1.13)	0.77(1.05)	0.87(0.87)	1.60(2.22)	1.99(2.89)
|F3|	0.77(0.83)	0.74(0.99)	0.87(0.88)	1.57(2.20)	2.03(2.80)
|F4|	0.75(0.73)	0.77(1.04)	0.87(0.88)	1.62(2.33)	1.96(2.72)
|F7|	0.78(0.81)	0.68(0.90)	0.80(0.81)	1.30(1.77)	1.67(2.27)
|F8|	0.77(0.66)	0.73(0.92)	0.84(0.87)	1.25(1.77)	1.45(2.05)
|Cz|	0.75(0.68)	0.74(0.94)	0.85(0.88)	1.51(2.27)	1.39(1.93)
|C3|	0.73(0.69)	0.74(0.98)	0.85(0.93)	1.24(1.70)	1.60(2.27)
|C4|	0.71(0.75)	0.79(1.03)	0.84(0.94)	1.26(1.78)	1.44(2.33)
|Pz|	0.78(0.75)	0.65(0.76)	0.80(0.84)	1.19(1.74)	1.50(2.23)
|P3|	0.78(0.75)	0.66(0.79)	0.81(0.95)	1.13(1.60)	1.71(2.78)
|P4|	0.76(0.81)	0.69(0.77)	0.83(0.91)	1.14(1.63)	1.51(2.26)
|T3|	0.71(0.57)	0.76(0.93)	0.80(0.96)	1.32(1.68)	1.41(1.98)
|T4|	0.84(0.87)	0.77(0.89)	0.82(0.95)	1.36(1.83)	1.46(2.15)
|T5|	0.71(0.67)	0.71(1.06)	0.85(1.27)	1.06(1.58)	1.23(1.67)
|T6|	0.75(0.73)	0.83(0.92)	0.95(1.19)	1.39(2.10)	1.42(1.81)
|O1|	0.85(1.35)	0.68(0.78)	0.80(1.22)	1.17(1.65)	1.35(1.97)
|O2|	0.65(0.68)	0.61(0.65)	0.79(0.96)	1.04(1.35)	1.33(1.91)

## Data Availability

Data are available from corresponding upon request.

## Appendix A

**Table A1:** The correlations between BDI-II, BAI, and z-scores in the MDD group.

		BDI-II (Depression)			BAI (Anxiety)		
		Cognitive Depression	Somatic Depression	Total Depression Score	Cognitive Anxiety	Somatic Anxiety	Total Anxiety Score
delta	Fz	−0.09	−0.11	−0.10	−0.06	−0.09	−0.08
	F3	0.00	−0.11	−0.03	−0.03	−0.08	−0.06
	F4	−0.06	−0.15 *	−0.09	−0.08	−0.07	−0.09
	F7	−0.13	−0.11	−0.13	−0.07	−0.11	−0.09
	F8	−0.05	−0.08	−0.07	−0.13	−0.09	−0.12
	Cz	−0.03	−0.10	−0.05	−0.06	−0.10	−0.09
	C3	−0.05	−0.12	−0.07	−0.07	−0.11	−0.10
	C4	−0.03	−0.08	−0.05	−0.03	−0.06	−0.05
	Pz	−0.05	−0.11	−0.07	−0.06	−0.09	−0.09
	P3	−0.03	−0.09	−0.05	−0.06	−0.11	−0.09
	P4	−0.01	−0.11	−0.03	−0.04	−0.11	−0.08
	T3	−0.05	−0.14	−0.08	−0.16 *	−0.12	−0.16 *
	T4	−0.07	−0.14 *	−0.10	−0.08	−0.08	−0.09
	T5	0.01	−0.09	−0.02	−0.04	−0.04	−0.04
	T6	0.00	−0.10	−0.03	−0.06	−0.07	−0.07
	O1	0.03	−0.03	0.02	0.08	0.00	0.05
	O2	0.05	−0.07	0.03	−0.01	−0.05	−0.04
theta	Fz	−0.02	−0.05	−0.03	−0.02	−0.04	−0.03
	F3	−0.01	−0.05	−0.02	−0.02	−0.03	−0.03
	F4	−0.01	−0.08	−0.02	−0.01	−0.02	−0.02
	F7	−0.04	−0.05	−0.05	−0.02	−0.06	−0.05
	F8	0.03	−0.03	0.02	−0.01	0.00	−0.01
	Cz	0.02	−0.04	0.01	0.00	−0.03	−0.02
	C3	0.01	−0.04	0.00	−0.01	−0.03	−0.02
	C4	0.01	−0.05	0.00	0.00	−0.02	−0.01
	Pz	0.02	−0.05	0.00	0.00	−0.02	−0.01
	P3	0.04	−0.03	0.02	0.01	0.00	0.01
	P4	0.01	−0.05	0.00	−0.01	−0.01	−0.01
	T3	0.01	−0.02	0.00	−0.03	−0.08	−0.06
	T4	0.02	−0.05	0.01	−0.01	−0.02	−0.01
	T5	0.05	−0.04	0.03	0.00	0.05	0.03
	T6	0.01	−0.04	0.00	0.00	0.01	0.00
	O1	0.05	−0.01	0.04	0.03	0.00	0.02
	O2	0.05	−0.01	0.04	0.02	0.03	0.03
alpha	Fz	0.00	0.00	0.00	−0.02	0.05	0.01
	F3	−0.02	−0.03	−0.02	−0.03	0.03	0.00
	F4	−0.03	−0.06	−0.04	−0.03	0.05	0.01
	F7	0.00	−0.02	0.00	−0.03	0.04	0.00
	F8	0.02	0.00	0.02	−0.02	0.04	0.01
	Cz	−0.04	−0.05	−0.05	−0.06	−0.01	−0.04
	C3	−0.04	−0.06	−0.05	−0.06	−0.01	−0.04
	C4	−0.03	−0.07	−0.04	−0.06	0.00	−0.04
	Pz	−0.02	−0.05	−0.03	−0.04	0.02	−0.02
	P3	−0.01	−0.07	−0.03	−0.07	0.03	−0.02
	P4	0.00	−0.07	−0.02	−0.06	0.06	0.00
	T3	0.05	0.02	0.05	−0.04	0.02	−0.01
	T4	0.01	−0.05	−0.01	−0.04	0.03	−0.01
	T5	0.05	−0.01	0.04	−0.03	0.06	0.01
	T6	0.03	−0.03	0.02	−0.01	0.12	0.06
	O1	0.06	−0.01	0.05	0.03	0.08	0.06
	O2	0.08	0.00	0.07	0.05	0.12	0.10
beta	Fz	0.04	0.05	0.04	0.02	0.10	0.06
	F3	0.04	0.03	0.04	0.03	0.12	0.08
	F4	0.01	−0.01	0.01	0.01	0.11	0.06
	F7	0.05	0.05	0.06	0.02	0.08	0.05
	F8	0.09	0.06	0.09	0.02	0.10	0.07
	Cz	−0.01	0.02	0.00	0.00	0.09	0.05
	C3	0.04	0.03	0.04	0.03	0.13	0.09
	C4	0.01	−0.01	0.01	0.00	0.11	0.06
	Pz	0.02	0.01	0.02	0.04	0.16 *	0.11
	P3	0.05	0.03	0.05	0.05	0.19 **	0.13 *
	P4	0.04	0.02	0.04	0.05	0.17*	0.12
	T3	0.04	0.08	0.06	0.00	0.09	0.05
	T4	0.02	0.01	0.02	0.00	0.10	0.05
	T5	0.12	0.08	0.12	0.06	0.19 **	0.14 *
	T6	0.08	0.02	0.07	0.08	0.16 *	0.13 *
	O1	0.11	0.05	0.11	0.12	0.19 **	0.17 *
	O2	0.15 *	0.04	0.14 *	0.12	0.21 **	0.18 **
high-	Fz	0.07	0.11	0.08	0.06	0.11	0.09
beta	F3	0.08	0.10	0.09	0.07	0.12	0.10
	F4	0.04	0.08	0.06	0.06	0.12	0.10
	F7	0.11	0.11	0.12	0.04	0.13	0.09
	F8	0.14 *	0.13	0.15 *	0.08	0.15 *	0.12
	Cz	0.06	0.11	0.08	0.07	0.14 *	0.12
	C3	0.06	0.12	0.08	0.05	0.13	0.10
	C4	0.03	0.08	0.05	0.03	0.08	0.06
	Pz	0.05	0.08	0.06	0.04	0.16 *	0.11
	P3	0.05	0.09	0.07	0.05	0.13	0.10
	P4	0.07	0.12	0.08	0.04	0.15 *	0.11
	T3	0.03	0.17 *	0.07	0.03	0.06	0.05
	T4	0.04	0.11	0.06	0.07	0.09	0.09
	T5	0.14 *	0.17 *	0.16 *	0.12	0.18 **	0.16 *
	T6	0.07	0.09	0.08	0.06	0.15 *	0.11
	O1	0.10	0.08	0.11	0.05	0.11	0.09
	O2	0.07	0.06	0.07	0.01	0.11	0.06

* *p* < 0.05, ** *p* < 0.01. Note: BDI-II, Beck Depression Inventory-II; BAI, Beck Anxiety Inventory.
